# Predictive Models for Knee Pain in Middle-Aged and Elderly Individuals Based on Machine Learning Methods

**DOI:** 10.1155/2022/5005195

**Published:** 2022-09-26

**Authors:** Lu Liu, Min-min Zhu, Lin-lin Cai, Xiao Zhang

**Affiliations:** ^1^Department of Anesthesiology, The Affiliated Wuxi NO.2 People's Hospital of Nanjing Medical University, Wuxi, Jiangsu, China; ^2^Department of Anesthesiology, Wuxi NO.2 People's Hospital, Wuxi, Jiangsu, China

## Abstract

**Aim:**

This study used machine learning methods to develop a prediction model for knee pain in middle-aged and elderly individuals.

**Methods:**

A total of 5386 individuals above 45 years old were obtained from the National Health and Nutrition Examination Survey. Participants were randomly divided into a training set and a test set at a 7 : 3 ratio. The training set was used to create a prediction model, whereas the test set was used to validate the proposed model. We constructed multiple predictive models based on three machine learning methods: logistic regression, random forest, and Extreme Gradient Boosting. The model performance was evaluated by areas under the receiver (AUC), sensitivity, specificity, positive predictive value, and negative predictive value. Additionally, we created a simplified nomogram based on logistic regression for better clinical application.

**Results:**

About 31.4% (1690) individuals were with self-reported knee pain. The logistic regression showed that female gender (odds ratio [OR] = 1.28), pain elsewhere (OR = 4.64), and body mass index (OR = 1.05) were significantly associated with increased risk of knee pain. In the test set, the logistic regression (AUC = 0.71) showed similar but slightly higher accuracy than the random forest (AUC = 0.70), while the performance of the Extreme Gradient Boosting model was less reliable (AUC = 0.59). Based on mean decrease accuracy, the most important first five predictions were pain elsewhere, waist circumference, body mass index, age, and gender. Additionally, the most important first five predictions with the highest mean decrease Gini index were pain elsewhere, body mass index, waist circumference, triglycerides, and age. The nomogram model showed good discrimination ability with an AUC of 0.75 (0.73-0.77), a sensitivity of 0.72, specificity of 0.71, a positive predictive value of 0.45, and a negative predictive value of 0.88.

**Conclusion:**

This study proposed a convenient nomogram tool to evaluate the risk of knee pain for the middle-aged and elderly US population in primary care. All the input variables can be easily obtained in a clinical setting, and no additional radiologic assessments were required.

## 1. Introduction

Knee pain is estimated to affect about 35% of men and 62% of women over 40 years old, constituting a significant health threat worldwide [1]. Patients with knee pain usually experience reduced physical ability and poor life quality [2–4]. The disease burden of knee pain is increasing due to the aging population and limited preventive strategies. For middle-aged and elderly, knee osteoarthritis is the primary cause of knee pain [5]. Importantly, most knee joint diseases usually progress slowly but would eventually result in joint failure with pain and disability.

However, there lacks a close association between radiological alteration and the occurrence of pain, and it remains unclear at which stage the disease would cause knee pain [6]. Considering the significant individual and socioeconomic burden, attention has been paid to the early detection and prevention of knee pain [7]. Many studies have revealed risk factors for knee pain, such as elder age, female gender, and obesity. A better understanding of the risk factor could provide an insightful and cost-effective tool for identifying those with an increased risk of knee pain [8]. When high-risk individuals are identified, clinicians can offer them preventive strategies and change their lifestyles [9].

Although previous studies have proposed prediction models for joint pain or osteoarthritis [10–12], the small sample size and the clinical inapplicability make it difficult to apply to clinical practice. Therefore, this study sought to develop a risk prediction model for knee pain based on easily obtained demographics and laboratory biomarkers. Multiple machine learning methods (logistic regression, random forest, and Extreme Gradient Boosting) were applied, and we visualized the logistic regression model using a nomogram.

## 2. Methods

### 2.1. Study Population

The National Health and Nutrition Examination Survey (NHANES) is a program survey for the health and nutritional status of the US population. We analyzed two continuous NHANES surveys, including the 2001-2002 and the 2003-2004 surveys. Participants with demographic, anthropometry, and laboratory records were included in this study. We excluded those below 45 years old or without records of joint pain (*N* = 4366). A total of 5386 individuals were analyzed in this study.

### 2.2. Knee Pain Assessment

In the continuous NHANES survey, self-reported knee pain was obtained by questionnaires (detailed descriptions were provided at: http://wwwn.cdc.gov/Nchs/Nhanes/2001-2002/MPQ_B.htm). Participants were first asked the screening question: “Joint pain/aching/stiffness in past year?”. For those who answered ‘Yes', they would be subsequently asked the following questions: ‘Right/left shoulder affected?', ‘Right/left elbow affected?', ‘Right/left hip affected?', ‘Right/left wrist affected?', ‘Right/left knee affected?', ‘Right/left ankle affected?', ‘Right toes affected?', and ‘Right fingers/thumb affected?'. Based on the questionnaires, we identified patients with joint pain and the affected joints. We defined patients with knee pain as those who responded ‘Yes' to ‘Right/left knee affected?'. Additionally, pain in other areas (shoulder, elbow, hip, wrist, ankle, toes, or fingers) was defined as pain elsewhere.

### 2.3. Predictive Biomarkers

This study selected multiple predictive biomarkers associated with knee pain based on literature review and expert recommendations [13, 14]. All selected biomarkers can be easily obtained by inquiry, body measure, and blood test. Age (years), gender (male or female), race (Non-Hispanic White, Non-Hispanic Black, Mexican American, and others), education (Below high school, high school, and above high school), hypertension (yes or no), diabetes (yes or no), pain elsewhere (shoulder, elbow, hip, wrist, ankle, toes, or fingers/thumb), moderate activity (yes or no), vigorous activity (yes or no), smoking (yes or no), and drinking (yes or no) were collected by questionnaires. Body mass index (BMI) and waist circumference were obtained by body measure. Plasm levels of albumin (g/L), phosphorus (mg/dL), total calcium (mg/dL), triglycerides (mg/dL), cholesterol (mg/dL), and vitamin D (nmol/L), were also examined. Additionally, we calculated the estimated glomerular filtration rate (eGFR) by the Chronic Kidney Disease Epidemiology Collaboration equation [15].

### 2.4. Development and Validation of Prediction Model

Participants were randomly divided into a training set and a test set at a 7 : 3 ratio. The training set was used to create a prediction model, whereas the test set was used to validate the proposed model. Patients' characteristics in the training and testing set are shown in Table S1. The upmentioned variables were used as inputs, and we set the prevalence of knee pain as the outcome. We constructed multiple predictive models based on multiple machine learning methods, including logistic regression, random forest, and Extreme Gradient Boosting (XGBoost). Random forest is an ensemble learning method for data regression and classification based on a multitude of decision trees [16], whereas XGBoost is a scalable end-to-end tree boosting system. We showed the model performance by receiver operating characteristic curve and calculated the areas under the receiver (AUC) of the three models. Sensitivity, specificity, positive predictive value, and negative predictive value were also provided.

Moreover, we used gender, age, hypertension, diabetes, vitamin D, pain elsewhere, total calcium, waist circumference, and BMI to create a logistic regression-based prediction model. The prediction model was then visualized by a nomogram, which is more practical for clinical application. Each variable of the nomogram was assigned a preliminary score, and the total score could be accordingly calculated. Eventually, the total score would be converted to the probability of knee pain (0-100%).

### 2.5. Statistical Analysis

The missing variables were filled by the multivariate multiple imputation method. Continuous variables were presented as median (Q1, Q3) and compared by the Kruskal-Wallis test between groups, whereas the categorical variables were presented as percentages and compared by the chi-square test. We performed multivariate regression to investigate the association between the biomarkers and knee pain. Analyses were performed by R software (version 3.6.1). *P* < 0.05 as considered statistically significant.

## 3. Results

### 3.1. Study Population

Table 1 describes the characteristics of the study population. Among the 5386 individuals, 1690 (about 31.4%) had self-reported knee pain. Compared with the normal group, knee pain patients were more with gender sex, lower education, hypertension, diabetes, pain elsewhere, moderate activity, and vigorous activity. Also, knee pain patients showed higher BMI, waist circumference, and triglycerides but low vitamin D levels.

### 3.2. Multivariable Logistic Regression

We performed logistic regression on the included biomarkers. The results showed that female gender (OR = 1.28, 95% CI = 1.06 − 1.55), pain elsewhere (OR = 4.64, 95% CI = 3.98 − 5.43), and BMI (OR = 1.05, 95% CI = 1.02 − 1.08) were significantly associated with increased risk of knee pain (Table 2).

### 3.3. Performance of the Prediction Models

**Figure 1:**
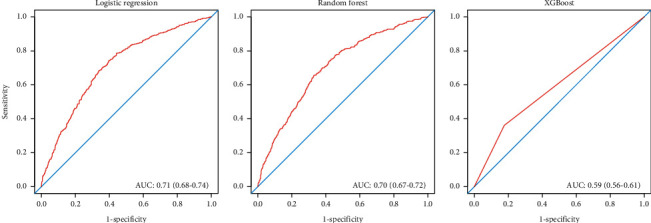
The receiver operating characteristic curves of the logistic regression, random forest, and XGBoost based on the test set.

We used age, gender, race, education, hypertension, diabetes, pain elsewhere, moderate activity, vigorous activity, smoking, drinking, BMI, waist circumference, albumin, phosphorus, total calcium, triglycerides, cholesterol, vitamin D, and eGFR as input variables. Three different models based on logistic regression, random forest, and XGBoost were created using the training set, respectively. Among the three models, logistic regression (AUC = 0.71, 95% CI = 0.68 − 0.74) showed similar but slightly higher accuracy than random forest (AUC = 0.70, 95% CI = 0.67 − 0.72), while the performance of the XGBoost model was less reliable (Figure 1).

**Figure 2:**
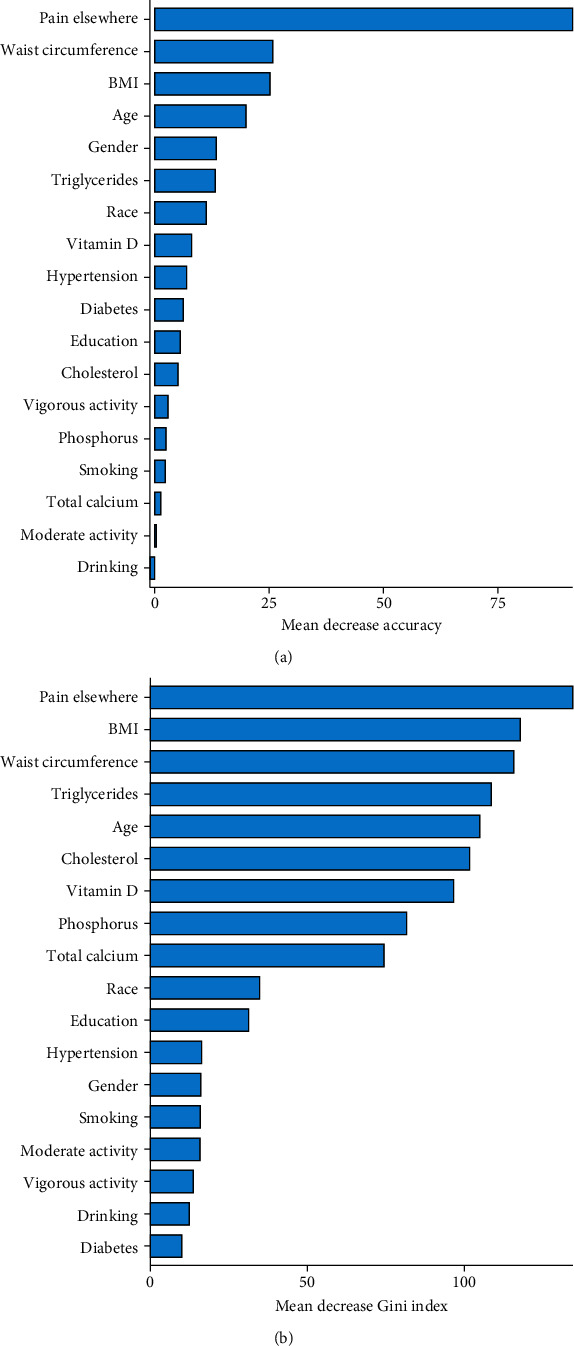
Variable importance of the random forest model evaluated by (a) mean decrease accuracy and (b) mean decrease Gini index.

The random forest model shows a sensitivity of 0.72, specificity of 0.61, a positive predictive value of 0.46, and a negative predictive value of 0.83. The variable importance of the random forest model is illustrated in Figure 2. The higher mean decrease accuracy and decrease Gini index suggested the more important role of a variable in knee pain. Based on mean decrease accuracy, the most important first five predictions were pain elsewhere, waist circumference, body mass index, age, and gender. Additionally, the most important first five predictions with the highest mean decrease Gini index were pain elsewhere, body mass index, waist circumference, triglycerides, and age. The logistic regression model shows a sensitivity of 0.71, a specificity of 0.64, a positive predictive value of 0.47, and a negative predictive value of 0.83.

**Figure 3:**
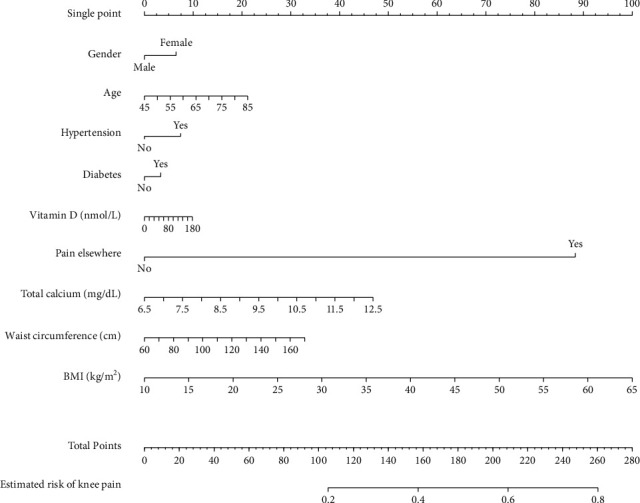
The prediction model based on logistic regression visualized by a nomogram.

Moreover, the prediction model based on logistic regression was visualized by a nomogram (Figure 3). Gender, age, hypertension, diabetes, vitamin D, pain elsewhere, total calcium, waist circumference, and BMI were input into the simplified nomogram. In the testing set, the nomogram model showed good discrimination ability with an AUC of 0.75 (0.73-0.77), a sensitivity of 0.72, specificity of 0.71, a positive predictive value of 0.45, and a negative predictive value of 0.88.

## 4. Discussion

Knee pain is closely associated with the middle-aged and elderly population and has become a major reason for early retirement. The NHANES I survey indicated that about 14.6% population reported knee pain [17]. It was reported that knee pain made about 20% of individuals with knee osteoarthritis retire earlier by eight years [18]. This study developed machine learning models to evaluate the risk of knee pain for the general US population. In the test set, random forest showed similar but slightly higher accuracy than logistic regression, while the performance of the XGBoost model was less reliable.

Many risk factors have been proposed for knee pain, such as age, female gender, obesity, and pain elsewhere. [8, 14, 19]. However, individuals with one or some risk factors might not experience knee pain, and a single risk factor alone was insufficient to evaluate the disease risk comprehensively. Therefore, several conventional risk factors were used as the input of the prediction models in this study. All the input variables can be measured easily in clinical practice, and no radiologic assessment was required in these models. Using easily available biomarkers without additional laboratory or radiologic examinations makes the prediction model simple to use and cost-effective. The nomogram model has a high negative predictive value (0.84) but a lower positive predictive value (0.47). Therefore, this prediction model is more suitable for identifying individuals with low knee pain risk.

The aging-induced joint pain is a multifactorial process involving numerous factors, such as cartilage thinning, muscle weakening, and proprioception reduction. Aging would also decline the capability of maintaining tissue homeostasis, thus causing an inadequate response to joint injury. We also observed that BMI and waist circumference were positively associated with a higher risk of knee pain [20]. Due to the population aging and the elevated obesity prevalence, knee pain was expected to be a growing health problem.

Besides aging and obesity, other biomarkers were also involved in the prediction models [8]. Pain elsewhere (shoulder, elbow, hip, wrist, ankle, toes, or fingers/thumb) was a significant biomarker for knee pain with an OR of 4.64 (95% CI = 3.98 − 5.43), which was consistent with previous studies [14, 18]. The association between joint pain and pain elsewhere might be attributed to the shared pathology or the progress of chronic pain syndrome [21]. Fernandes et al. [18] analyzed 1822 participants at risk for knee pain from the Nottingham community and followed the participants for 12 years. The results showed that pain elsewhere led to a 2.49-fold risk of knee pain [18]. In another prospective cohort study of 2982 people, the baseline pain other than the knee increased the risk of the new onset of knee pain but not for the progression from mild to severe [14].

Previous studies also proposed prediction models for joint pain or osteoarthritis [10–12]. Zhang et al. created a prediction model for radiographic knee osteoarthritis based on a 12-year retrospective community cohort (UK Nottingham cohort). Age, gender, BMI, family history, and joint injury were included in the prediction model (AUC = 0.70) [11]. Similarly, Kerkhof et al. [10] used age, gender, BMI, questionnaire variables, genetic scores, and radiographic signs to develop a prediction model for radiographic knee osteoarthritis based on Netherlands individuals aged 55 years and over (AUC = 0.79). Compared with previous prediction models, our model showed similar accuracy but was based on easily available biomarkers without additional laboratory or radiologic assessments. These advantages make it a simple-to-use and cost-effective tool suitable for primary care.

Still, some limitations should be motioned. First, the definition of knee pain was based on self-reported knee pain. A proportion of self-reported knee pain might be referred to as hips/spine pain instead of pain from the knee. Second, we tested the model performance in the internal set. However, we are unsure if the proposed knee pain prediction tool can be applied to other populations, such as the Chinese or European population. Third, the NHANES was a cross-sectional design which induces the inherent bias. Further validation and improvement were required in the following research. Fourth, although we input multiple variables in the model, many risk factors potentially remain. The investigation of additional biomarkers would improve the model performance.

## 5. Conclusion

This study proposed a convenient tool to evaluate the risk of knee pain for the middle-aged and elderly US population in primary care. All the input variables can be easily obtained in a clinical setting, and no additional radiologic assessments were required. In the internal validation, the nomogram model showed reliable performance with an AUC of 0.72.

## Figures and Tables

**Table 1:** The characteristics of the study population.

	Knee pain	No knee pain	*P*
*N*	1690	3696	
Age	64.0 (54.0, 74.0)	64.0 (53.0, 74.0)	0.202
Gender (female), *n* (%)	962 (56.9%)	1758 (47.6%)	< 0.001
Race, *n* (%)			0.383
Non-Hispanic white	977 (57.8%)	2150 (58.2%)	
Non-Hispanic black	320 (18.9%)	643 (17.4%)	
Mexican American	296 (17.5%)	658 (17.8%)	
Others	97 (5.7%)	245 (6.6%)	
Education, *n* (%)			0.029
Below high school	591 (35.0%)	1223 (33.1%)	
High school	424 (25.1%)	855 (23.1%)	
Above high school	675 (39.9%)	1618 (43.8%)	
Hypertension (yes), *n* (%)	917 (54.3%)	1648 (44.6%)	< 0.001
Diabetes (yes), *n* (%)	305 (18.0%)	549 (14.9%)	0.003
Pain elsewhere (yes), *n* (%)	1192 (70.5%)	1247 (33.7%)	< 0.001
Moderate activity (yes), *n* (%)	683 (40.4%)	1663 (45.0%)	0.002
Vigorous activity (yes), *n* (%)	255 (15.1%)	734 (19.9%)	< 0.001
Smoking (yes), *n* (%)	920 (54.4%)	2014 (54.5%)	0.994
Drinking (yes), *n* (%)	315 (18.6%)	638 (17.3%)	0.234
BMI (kg/m^2^)	29.1 (25.8, 33.3)	27.1 (24.1, 30.6)	< 0.001
Waist circumference (cm)	102.5 (93.5, 111.8)	98.2 (89.4, 107.3)	< 0.001
Albumin (g/L)	42.0 (40.0, 44.0)	42.0 (40.0, 44.0)	0.002
Phosphorus (mg/dL)	3.7 (3.4, 4.1)	3.7 (3.4, 4.0)	0.075
Total calcium (mg/dL)	9.5 (9.2, 9.7)	9.5 (9.2, 9.7)	0.590
Triglycerides (mg/dL)	128.0 (89.0, 184.8)	123.0 (85.0, 179.0)	0.018
Cholesterol (mg/dL)	205.0 (179.0, 234.8)	205.0 (180.0, 233.0)	0.608
Vitamin D (nmol/L)	55.7 (39.7, 70.6)	58.1 (43.4, 72.9)	< 0.001
eGFR (ml/min/1.73m^2^)	77.9 (20.6)	78.4 (20.6)	0.410

BMI: body mass index; eGFR: estimated glomerular filtration rate.

**Table 2:** Multivariable logistic regression.

	Odds ratio	95% CI	*P*
Age	1.01	1.00-1.02	0.105
Gender (female)	1.28	1.06-1.55	0.011
Race			
Non-Hispanic white	*Ref*		
Non-Hispanic black	1.15	0.91-1.45	0.249
Mexican American	1.02	0.81-1.28	0.889
Others	0.91	0.65-1.26	0.563
Education			
Below high school	*Ref*		
High school	1.06	0.86-1.31	0.588
Above high school	0.95	0.78-1.15	0.598
Hypertension (yes)	1.11	0.95-1.31	0.198
Diabetes (yes)	0.91	0.73-1.12	0.376
Pain elsewhere (yes)	4.64	3.98-5.43	< 0.001
Moderate activity (yes)	1.04	0.89-1.22	0.617
Vigorous activity (yes)	0.90	0.72-1.12	0.338
Smoking (yes)	1.12	0.96-1.31	0.158
Drinking (yes)	0.86	0.70-1.05	0.152
BMI (kg/m^2^)	1.05	1.02-1.08	< 0.001
Waist circumference	1.00	0.99-1.02	0.471
Albumin (g/L)	1.01	0.98-1.04	0.584
Phosphorus (mg/dL)	0.99	0.85-1.15	0.850
Total calcium (mg/dL)	1.01	0.82-1.24	0.927
Triglycerides (mg/dL)	1.00	1.00-1.00	0.470
Cholesterol (mg/dL)	1.00	1.00-1.00	0.674
Vitamin D (nmol/L)	1.00	0.99-1.00	0.438
eGFR (ml/min/1.73m^2^)	1.00	1.00-1.01	0.457

BMI: body mass index; eGFR: estimated glomerular filtration rate.

## Data Availability

The data in this study can be obtained from https://www.cdc.gov/nchs/nhanes/index.htm

## Supplementary Materials

Supplementary Materials Table S1. Characteristic between the training and testing set.
