# Supplementary material for: Predictive Models for Knee Pain in Middle-Aged and Elderly Individuals Based on Machine Learning Methods
Source: Comput Math Methods Med. 2022 Sep 26;2022:5005195. doi: 10.1155/2022/5005195 (PMC9529423; doi:10.1155/2022/5005195)
Supplement: Supplementary Materials — Table S1. Characteristic between the training and testing set. [file 5005195.f1.docx]

**STable 1**. Characteristic between the training and testing set.

|  | **Testing set** | **Training set** | ***P*** |
| --- | --- | --- | --- |
| N | 1616 | 3770 |  |
| Knee pain (Yes), n (%) | 507 (31.4%) | 1183 (31.4%) |  |
| Age | 64.0 (53.0, 74.0) | 64.0 (53.0, 74.0) | 0.349 |
| Gender (Female), n (%) | 792 (49.0%) | 1928 (51.1%) | 0.160 |
| Race, n (%) |  |  | 0.236 |
| Non-Hispanic White | 936 (57.9%) | 2191 (58.1%) |  |
| Non-Hispanic Black | 312 (19.3%) | 651 (17.3%) |  |
| Mexican American | 270 (16.7%) | 684 (18.1%) |  |
| Others | 98 (6.1%) | 244 (6.5%) |  |
| Education, n (%) |  |  | 0.279 |
| Below high school | 519 (32.1%) | 1295 (34.4%) |  |
| High School | 395 (24.4%) | 884 (23.4%) |  |
| Above high school | 702 (43.4%) | 1591 (42.2%) |  |
| Hypertension (Yes), n (%) | 735 (45.5%) | 1830 (48.5%) | 0.042 |
| Diabetes (Yes), n (%) | 259 (16.0%) | 595 (15.8%) | 0.854 |
| Pain elsewhere (Yes), n (%) | 720 (44.6%) | 1719 (45.6%) | 0.500 |
| Moderate activity (Yes), n (%) | 708 (43.8%) | 1638 (43.4%) | 0.828 |
| Vigorous activity (Yes), n (%) | 317 (19.6%) | 672 (17.8%) | 0.129 |
| Smoking (Yes), n (%) | 892 (55.2%) | 2042 (54.2%) | 0.504 |
| Drinking (Yes), n (%) | 301 (18.6%) | 652 (17.3%) | 0.256 |
| BMI (kg/m^2^) | 27.8 (24.7, 31.5) | 27.7 (24.6, 31.5) | 0.507 |
| Waist circumference (cm) | 99.7 (91.3, 109.2) | 99.4 (90.5, 108.9) | 0.380 |
| Albumin (g/L) | 42.0 (40.0, 44.0) | 42.0 (40.0, 44.0) | 0.547 |
| Phosphorus (mg/dL) | 3.7 (3.4, 4.1) | 3.7 (3.4, 4.1) | 0.975 |
| Total calcium (mg/dL) | 9.5 (9.2, 9.7) | 9.5 (9.2, 9.7) | 0.301 |
| Triglycerides (mg/dL) | 125.0 (85.0, 185.0) | 124.5 (86.0, 180.0) | 0.983 |
| Cholesterol (mg/dL) | 205.0 (178.0, 232.0) | 205.0 (180.0, 234.0) | 0.366 |
| Vitamin D (nmol/L) | 57.2 (42.0, 72.9) | 56.3 (42.0, 70.6) | 0.424 |
| eGFR (ml/min/1.73m^2^) | 78.3 (20.2) | 78.3 (20.7) | 0.926 |

BMI: body mass index; eGFR: estimated glomerular ﬁltration rate.
